# Diffusion and division: A spatial analysis of surrogacy policy determinants in the United States

**DOI:** 10.1016/j.ssmph.2025.101876

**Published:** 2025-10-30

**Authors:** Jingjing Gao, Muinat Abolore Idris, Gabriela A. Gallegos, Bryan Colby Griffin, Sharon V. Munroe, Jason H. Windett

**Affiliations:** a Department of Management, Policy & Community Health, University of Texas Health Science Center at Houston School of Public Health, El Paso, TX, USA; b Department of Health Promotion and Behavioral Sciences, School of Public Health, University of Texas Health Science Center at Houston, Houston, TX, USA; c Department of Health Promotion and Behavioral Sciences, School of Public Health, University of Texas Health Science Center at Houston, Houston, TX, Austin, TX, USA; d School of Data Science, Department of Political Science and Public Administration, Public Policy Ph.D. Program, University of North Carolina at Charlotte, Charlotte, NC, 28262, USA

## Abstract

**Background:**

Advancements in reproductive technologies have made surrogacy an increasingly attractive option for individuals and couples facing fertility challenges. In 2022, the global commercial surrogacy market was valued at approximately $14 billion, with the United States contributing a significant share due to its robust healthcare and legal infrastructure. However, surrogacy policy in the United States remains highly fragmented—some states have permissive legal frameworks, others prohibit surrogacy entirely, and many have no formal statutes. This study examines how states’ political ideology, religiosity, and socioeconomic factors influence the adoption of surrogacy policies across the United States.

**Methods:**

We conducted a spatial analysis using data from the United States Census Bureau, World Population Review, and the United States Surrogacy Law Map. States were categorized based on the permissiveness of their surrogacy policies. Spatial lag regression model and ordered logistic regression models were employed to assess associations between policy status and independent variables, including religiosity, political affiliation, income, and racial demographics.

**Results:**

Moran's I indicated significant positive spatial clustering of surrogacy law permissiveness across states (I = 0.206, p < 0.05), suggesting geographic diffusion of policy environments. Spatial lag regression results showed that higher religiosity was associated with more restrictive policies, although this effect attenuated after adjusting for socioeconomic and political factors. Ordered logistic regression models confirmed these associations while explicitly accounting for the ordinal outcome structure: higher religiosity significantly decreased the odds of permissive policies (Model 1: β = −0.124, p < 0.05), whereas racial diversity predicted greater policy permissiveness. In fully adjusted models, the percentage of White, Black, and Hispanic residents remained positive and significant predictors of permissive surrogacy laws, while religiosity trended negative but fell just short of statistical significance (p = 0.07).

**Conclusion:**

This study demonstrates that a combination of religiosity, racial composition, and spatial proximity to like-minded states shapes state-level surrogacy policy in the United States. Higher religious adherence is linked to more restrictive policies, while a greater proportion of White residents correlates with increased permissiveness when broader structural factors are considered. The findings underscore the importance of accounting for sociopolitical and geographic context in reproductive policy analysis. To promote equitable access to assisted reproductive technologies, public health efforts and legal reforms must consider these underlying sociocultural and spatial dynamics.

## Highlights

• U.S. surrogacy laws cluster geographically, with restrictive policies in the South and Midwest.
• Spatial models show religiosity and racial makeup shape policy permissiveness.
• Results reveal inequities in access to reproductive care, urging targeted legal interventions.

## 1 Introduction

Surrogacy has emerged as a critical reproductive option for individuals and couples experiencing infertility, same-sex couples, and those unable to carry a pregnancy to term. With continued advancements in assisted reproductive technologies (ART), the global surrogacy market was valued at over $14 billion in 2022. The United States has played a leading role due to its sophisticated medical infrastructure, demand for reproductive services, and varied legal frameworks (Danielowski, 2023; Joslin, 2020; Ko, 2025). Despite technological progress and growing demand for surrogacy, their legal regulation remains uneven across the United States. (Allen, 2018; Kalantry, 2022; Mohapatra, 2015; Woodward et al., 2020). Surrogacy, a legally complex and ethically debated form of ART, presents unique policy challenges that intersect with broader questions of bodily autonomy, family structure, and moral values. While some states support commercial and altruistic surrogacy arrangements through explicit statutes, others prohibit such practices altogether or remain silent, resulting in legal ambiguity and unequal access to care across the United States (Feldman, 2018; Horsey, 2023; 10.13039/501100023931Perkins et al., 2017; White et al., 2021). Policies vary on how legal parentage is assigned—some prioritize the intent of the parties, while others emphasize biological or gestational connections (Feldman, 2018; Joslin, 2020; Mulligan, 2024). This affects the legal status of both surrogates and intended parents. Additionally, states set different requirements for who can be a surrogate or an intended parent, impacting who can access surrogacy as a family-building choice (Feldman, 2018; Joslin, 2020). While there is an ongoing debate about whether uniform surrogacy laws are desirable or achievable, the lack of consistency between states leads to cross-state travel and legal uncertainty (Beilby et al., 2024; Feldman, 2018; Joslin, 2020; Perkins et al., 2017).

This legal fragmentation has profound implications for public health, equity, and reproductive justice. Individuals in restrictive states may be forced to travel out-of-state, endure legal uncertainty, or abandon surrogacy as a choice altogether, exacerbating disparities in access to family-building resources (Beilby et al., 2024; Joslin, 2020; Perkins et al., 2017). Understanding the determinants of state-level surrogacy policy is therefore essential to advancing reproductive autonomy and ensuring equitable access to care.

Prior studies suggest that social values and political ideologies shape reproductive health policy broadly (A. Kulczycki, 2007a, 2007b; McDonald & Utomo, 2009), but the drivers of surrogacy policy adoption remain understudied. Research has documented the role of political ideology in shaping reproductive health policies, including access to abortion, in vitro fertilization, and contraceptive services (Jones & Jerman, 2017). Also, states with conservative political leadership are more likely to adopt restrictive reproductive laws (Miller & Gomez, 2019; Rothstein, 2020). Furthermore, findings in the health policy literature show that sociocultural values—particularly religiosity—shape attitudes toward nontraditional family formation and bioethical innovations (Kimport, 2019). Religiosity, in particular, has also been shown to influence state-level attitudes toward reproductive rights, including abortion and in vitro fertilization (IVF), yet its relationship with surrogacy legislation is less well understood. Higher religiosity is consistently linked to more restrictive or negative views on reproductive rights, while lower religiosity or lack of religious affiliation is associated with greater support for these rights (Aburto-González et al., 2022; Chu-Nguyen et al., 2024; Yorulmaz, 2023). Similarly, racial-demographic context may shape the social and legislative environment in which surrogacy policies evolve (A. Abramowitz & Jennifer McCoy, 2019a; Engelhardt, 2020; Peterson & Westwood, 2020).

However, surrogacy policy remains an underexamined domain, with limited empirical research analyzing its geographic and demographic determinants at the state level. Spatial diffusion theory posits that policy adoption is often influenced not only by internal state characteristics but also by neighboring jurisdictions, through mechanisms such as policy learning, emulation, and normative pressure (Berry & Berry, 2018, pp. 253–297; Mitchell, 2018; Shipan & Volden, 2008). This is particularly relevant in domains like reproductive health, where cultural and legal norms vary regionally. Recent public health research has increasingly adopted spatial analytic techniques to examine policy clustering and regional inequities (Arcaya et al., 2020; Fayet et al., 2020; Miller & Vasan, 2021), but few studies have applied these tools to reproductive rights, particularly surrogacy.

This study builds on and extends this literature by using spatial regression techniques to investigate how religiosity, racial demographics, political control, and socioeconomic conditions are associated with state-level surrogacy law permissiveness. In doing so, it addresses a critical gap in understanding the sociopolitical geography of reproductive access in the United States. This study aims to fill these gaps by identifying the sociopolitical and demographic factors associated with the adoption of surrogacy policies across the United States. We use spatial regression models to examine how state-level variation in religiosity, race, income, education, and political control predicts surrogacy law permissiveness, accounting for the spatial clustering of policy environments. By integrating political science, public health, and spatial analysis, our study provides a comprehensive assessment of how state characteristics influence access to reproductive technologies in the United States and offers insight into how policy diffusion may perpetuate or mitigate structural inequities.

## 2 Methods: data sources

### 2.1 Surrogacy policy Measurement

State-level surrogacy policy information was compiled from the interactive map provided by Creative Family Connections (2024), which offers up-to-date, comprehensive summaries of surrogacy laws and regulations across the United States. This dataset categorizes each state by the presence or absence of specific surrogacy legislation, enabling comparative analysis of policy adoption patterns. The resource is publicly accessible and regularly updated, making it a reliable source for examining the legal landscape influencing reproductive services. States were classified into three categories: restrictive, neutral, and permissive. Specifically, we explain that classification was based on legal provisions regarding compensated gestational surrogacy contracts, parentage recognition, and statutory clarity. States were categorized into three levels of surrogacy policy permissiveness using the Creative Family Connections Surrogacy Law Map (2024). “Restrictive” states (e.g., Michigan, Louisiana) prohibit compensated surrogacy contracts or criminalize participation. “Neutral” states (e.g., Pennsylvania, North Carolina) lack explicit statutory guidance, leaving surrogacy governed primarily by case law or judicial discretion. “Permissive” states (e.g., California, Illinois) explicitly allow gestational surrogacy contracts and recognize intended parents through pre-birth parentage orders. This classification system provides a standardized, cross-state measure of policy environments relevant to access and legal certainty for intended parents and surrogates. In total, 50 U.S. states were classified according to their surrogacy law environment. Six states (12 %) were coded as **restrictive**, meaning that they prohibit compensated surrogacy contracts, impose criminal penalties, or explicitly deny legal recognition to intended parents (e.g., Michigan, Nebraska). Thirty states (60 %) were coded as **neutral**, reflecting the absence of explicit statutory regulation, where outcomes are determined by judicial interpretation or case-by-case rulings (e.g., Pennsylvania, North Carolina). Fourteen states (28 %) were coded as **permissive**, explicitly allowing gestational surrogacy contracts, including compensated arrangements, and granting pre-birth or streamlined parentage orders to intended parents (e.g., California, Illinois). These categories reflect substantive policy characteristics, including statutory clarity, enforceability of contracts, and recognition of parental rights.

### 2.2 Political environment measurement

Political context at the state level was characterized by two key variables: The governor's political party affiliation and the party control of state legislatures. Governor affiliation was classified as Democratic, Republican, or Other, based on the most recent data from Ballotpedia and the National Governors Association (Association, 2025; Ballotpedia, 2025), which compiles up-to-date information on governors' party membership. State legislative control was coded according to data from the National Conference of State Legislatures (NCSL), which tracks the partisan composition of both chambers in state legislatures. For this study, states were categorized as Democratic-controlled, Republican-controlled, or divided government, reflecting whether a single party held a majority in both legislative chambers and the governorship. These political variables provide essential contextual information, as party control has been shown to influence policy adoption and regulatory environments, particularly reproductive and family law policies. Incorporating these measures allows for a comprehensive analysis of how political governance structures correlate with surrogacy policy adoption across states.

### 2.3 State religiosity environment measurement

State-level religiosity was assessed using data from the *Pew Research Center's Religious Landscape Study* (2024), which provides comprehensive survey-based estimates of religious attitudes and behaviors across the United States. The measure includes indicators such as the percentage of adults who say religion is very important in their lives, attend services at least weekly, pray daily, and believe in God with absolute certainty. These variables were combined to generate a composite religiosity index, reflecting the cultural and social significance of religion within each state. This index reflects the overall intensity of religious commitment in each state but does not differentiate between specific religious traditions. Data were accessed from: https://www.pewresearch.org/religious-landscape-study/(Protestants, 2015; Smith et al., 2025).

### 2.4 State-level demographic and economic indicators for surrogacy policy analysis

State-level demographic, socioeconomic, and economic data were compiled from publicly available sources to characterize factors potentially influencing surrogacy policy adoption. Population size and density data were obtained from the United States Census Bureau's American Community Survey (ACS). Urban and rural population distributions were derived from the United States Census urban-rural classification. Educational attainment percentages were based on ACS estimates of adults with college degrees or higher. Median household income and poverty rates were also sourced from ACS data. Economic development indicators, including GDP per capita and unemployment rates, were collected from the United States Bureau of Economic Analysis (BEA) and the Bureau of Labor Statistics (BLS). These combined datasets provide a comprehensive overview of state-level contexts relevant to surrogacy policy analysis.

### 2.5 Statistical methods and software

All statistical analyses were conducted using a combination of spatial and regression techniques to account for geographic dependence in surrogacy policy adoption across the United States. Global spatial autocorrelation was assessed using Moran's I, calculated with a first-order Queen contiguity weights matrix to detect clustering in surrogacy law permissiveness (Janatabadi & Ermagun, 2024; Suryowati et al., 2018). Based on the presence of significant spatial autocorrelation, we applied spatial lag regression models (SAR) to estimate the influence of state-level covariates while adjusting for spatial dependence in the outcome variable (Gao et al., 2020; Lambert et al., 2010; Xu et al., 2020). This study treated policy permissiveness as continuous in order to estimate spatial lag dependence, consistent with prior spatial econometric practice. To directly model the ordinal structure, we now include ordered logistic regression models. This addition ensures that results are interpretable as the odds of being in a more permissive policy category, directly addressing the concern about ordinal categorical outcomes (Fullerton, 2009; Torres-Reyna, 2012). Nested models were used to sequentially introduce religiosity, racial demographics, and political and socioeconomic factors. Variance inflation factors (VIFs) were calculated to assess multicollinearity among predictors. Data cleaning and descriptive statistics were also conducted in Python 3. Visualizations, including policy distribution maps and spatial clustering outputs, were created using ArcGIS Pro (version 3.2) (Esri, 2024). All spatial econometric modeling (package *spreg*) and diagnostics were performed using Python 3 (Anselin & Rey, 2022; Rey, 2015; Rey & Anselin, 2009). Ordered logistic regression (*ologit*) was conducted by Stata 18.5 (StataCorp, 2024).

## 3 Results

### 3.1 Descriptive statistics

Table 1 presents descriptive statistics for all variables included in the analysis. The outcome variable, *surrogacy policy*, is categorized as restrictive (12 %), neutral (60 %), or permissive (28 %) among the 50 states. On average, 54.7 % of residents identified as religious, and the racial composition across states showed a predominance of White residents (M = 74.5 %, SD = 11.7), followed by Black (M = 10.9 %, SD = 9.5) and Hispanic populations (M = 10.9 %, SD = 9.8). States had a median household income of $61,297 (SD = $10,427), and an average unemployment rate of 4.2 % (SD = 0.83). Urban populations constituted 72.3 % (SD = 16.2) of residents on average, and approximately one-third of residents held a college degree (M = 31.2 %, SD = 5.7). Political indicators show near-equal distribution between Democratic and Republican affiliations and party control. These summary statistics reflect wide variability across the United States in sociodemographic, economic, and political characteristics, justifying the need for spatial regression methods to capture geographic dependencies and multilevel variation in surrogacy policies (see Table 2).

**Table 1:** Descriptive statistics of study variables (N = 50).

Variable	Mean (SD)	Median	Min	Max	Frequency (%)
Surrogacy:					
Restrictive					6 (12 %)
Neutral					30 (60 %)
Permissive					14 (28 %)
Religious (%)	54.70 (10.74)	54.00	33.0	77.0	
White (%)	74.52 (11.65)	74.75	48.0	93.8	
Black (%)	10.87 (9.50)	7.85	0.3	37.6	
Hispanic (%)	10.87 (9.84)	8.65	1.2	48.8	
Political Affiliation		2.00	1.0	2.0	
Party Control		2.00	1.0	2.0	
Median Income ($)		59,408	44,171	84,801	
Population	6,550,755.48 (7,389,284.95)	4,558,234	578,759	39,512,223	
Density (per sq mile)	202.53 (265.05)	106.00	1.3	1215.0	
Urban Population (%)	72.32 (16.17)	73.00	38.9	95.0	
College Degree (%)	31.24 (5.74)	30.00	20.7	43.5	
Unemployment Rate (%)	4.21 (0.83)	4.10	2.8	6.5

**Table 2:** Moran's I test for spatial autocorrelation of surrogacy laws.

Statistic	Value
Moran's I	0.206
p-value (normal test)	0.020
Spatial Method	Queen Contiguity (1st order)
Number of Units	50
Spatial Islands	Alaska, Hawaii

### 3.2 Spatial clustering of surrogacy laws across United States

Global spatial autocorrelation was assessed using Moran's I to determine whether surrogacy law permissiveness exhibited geographic clustering across the United States. Preliminary diagnostics using Moran's I indicated statistically significant positive spatial autocorrelation in surrogacy permissiveness (Moran's I = 0.206, *p* = 0.020), suggesting that neighboring states tend to adopt similar levels of legal permissiveness. This violates the assumption of independent observations underlying traditional ordinary least squares (OLS) regression and supports the use of spatial modeling techniques. Two states (Alaska and Hawaii) were identified as spatial “islands” with no contiguous neighbors and were flagged in the diagnostic output.

**Fig. 1:**
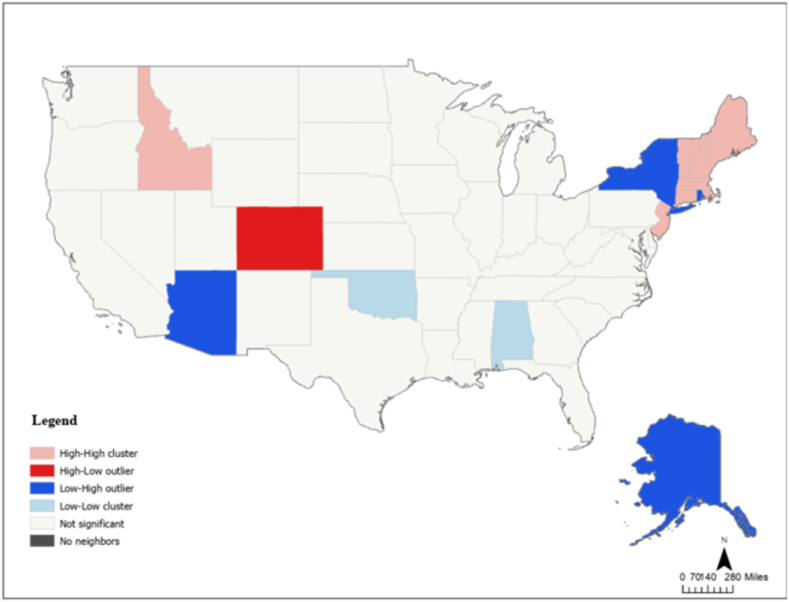
Geographic clusters and outliers in surrogacy policy restrictiveness: A Local Moran's I analysis of the United States.

To identify spatial clusters and outliers in state surrogacy policy restrictiveness, we conducted a Local Moran's I (Anselin LISA) analysis using 8-nearest-neighbor spatial weights in ArcGIS Pro. The analysis revealed several statistically significant spatial patterns (Fig. 1): 1) High-High clusters — states with restrictive surrogacy laws surrounded by similarly restrictive neighbors — were identified in the Mountain West (e.g., Idaho) and the Northeast (e.g., Massachusetts, Connecticut, and New York); 2) Low-Low clusters, indicating permissive states surrounded by similarly permissive neighbors, were not observed; 3) High-Low outliers — restrictive states surrounded by more permissive neighbors — included Colorado, suggesting a unique policy stance compared to its neighbors; 4) Low-High outliers, such as Arizona, Alaska, and New York, represented permissive states surrounded by more restrictive neighbors, possibly indicating progressive divergence or recent policy shifts; 5) The majority of states did not exhibit statistically significant spatial clustering, suggesting that while regional clustering exists, surrogacy policy diffusion is not uniform across the United States. These findings underscore important spatial dependencies in reproductive policy adoption and may reflect regional legal, cultural, or political influences shaping surrogacy regulation.

**Fig. 2:**
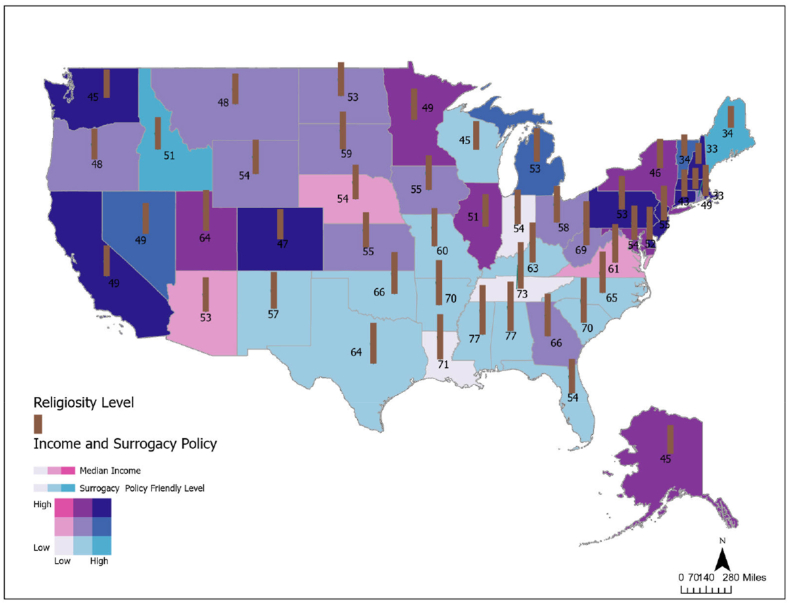
Geographic Variation in Surrogacy Policy Friendliness Across United States by Income, and Religiosity (brown bars) Note: Religiosity index ranges from 1 to 100.

Additionally, Fig. 2 visually shows the spatial distribution of surrogacy policy permissiveness level across the United States, which is represented by the surrogacy policy-friendly level in the map, revealing clear regional patterns and associations with both median income and religiosity. States in the Northeast and along the West Coast tend to have more surrogacy-friendly policies, depicted by dark blue shades, and are often characterized by higher median incomes and lower levels of religiosity. In contrast, states like Louisiana and Tennessee exhibit lower surrogacy policy friendliness, higher religiosity (represented by taller brown bars), and generally lower median incomes (minimal or no pink overlay). This suggests an inverse relationship between religiosity and surrogacy policy friendliness, where more religious states tend to maintain more restrictive surrogacy laws. Conversely, a positive association appears between income and policy friendliness, with higher-income states more likely to support permissive surrogacy regulations. These findings highlight the influence of both cultural and economic factors on the legal landscape of assisted reproductive technologies in the United States, with more progressive, affluent, and less religious regions offering greater support for surrogacy arrangements.

### 3.3 Modeling the sociopolitical and spatial determinants of surrogacy policy: assessment of multicollinearity among predictors

Surrogacy policy in the United States is shaped not only by within-state characteristics but also by regional diffusion, legal mimicry, and sociopolitical clustering. To account for this geographic interdependence, we employed spatial regression models, which explicitly model spatial autocorrelation in the outcome variable. We implemented the spatial lag model (SAR), which incorporates a spatially lagged dependent variable (W_surrogacy) as a predictor, allowing us to estimate both the direct effects of state-level covariates and the influence of neighboring states’ policy environments. This approach is theoretically appropriate given that policy innovation—especially in reproductive and health domains—is often shaped by geographic proximity, shared institutional norms, and interstate networks.

By modeling spatial dependence directly, our estimates avoid biased standard errors and better capture the true structure of policy diffusion in the United States context. To systematically examine the association between religiosity and surrogacy law permissiveness, we employed a stepwise regression strategy using a series of nested spatial lag models. This multilevel approach allowed us to isolate the unique contribution of religiosity and assess its robustness as additional explanatory variables were introduced.

Model 1 included religiosity as the sole predictor, serving as a baseline model to estimate its bivariate relationship with surrogacy permissiveness. This model reflects theoretical expectations that moral or cultural values—often proxied by religious adherence—shape reproductive policy.

Model 2 added racial composition variables (percent White, Black, and Hispanic residents) to examine whether the effect of religiosity is moderated or confounded by demographic structure. Including race allows us to test whether religiosity operates similarly across diverse population contexts or whether its effects are contingent upon racial majority status or diversity.

Model 3 examines whether religiosity's effect on surrogacy law permissiveness varies by racial composition. We modeled an interaction between the proportion of White residents and religious adherence.

Model 4 incorporated the full set of political, socioeconomic, and structural covariates, including income, education, urbanization, unemployment, and partisan control. This final model tests whether the observed relationship between religiosity (and race) and surrogacy policy persists when accounting for broader structural and political conditions known to influence policy adoption. Together, this progression from simple to fully adjusted models provide a transparent and theoretically grounded approach for identifying robust predictors of surrogacy permissiveness while controlling for potential confounding and spatial dependence.

To assess potential multicollinearity among predictors in the IVF policy model, variance inflation factors (VIFs) were calculated (see Table 3). Most variables showed VIF values well below the conventional threshold of 5, suggesting acceptable levels of multicollinearity. The highest VIFs were observed for White Percent (4.76), Hispanic Percent (4.57), and College Degree Percent (4.54), indicating moderate correlation with other predictors but still within an interpretable range. The intercept exhibited a very high VIF (687.79), which is expected and does not indicate multicollinearity among substantive variables. Overall, multicollinearity is not a major concern, and all predictors were retained in the model.

**Table 3:** Multicollinearity diagnostics: Variance inflation factors (VIFs) for predictors of surrogacy policy.

Variable	VIF
Constant (Intercept)	687.79
Political Affiliation	1.83
Party Control	2.91
Population	1.78
Density	1.86
Urban Percent	2.52
College Degree Percent	4.54
Median Income	3.95
White Percent	4.76
Black Percent	4.18
Hispanic Percent	4.57
Unemployment Rate	2.24

### 3.4 Spatial regression results: associations between religiosity, race, and surrogacy policy permissiveness

The spatial regression models (Table 4) reveal important associations between state-level characteristics and surrogacy policy permissiveness across the United States, accounting for spatial dependence between neighboring states (see Table 2). In Model 1, which includes only religiosity, higher religious adherence is significantly associated with more restrictive surrogacy laws (*β* = −0.024, *p* < 0.05). This suggests that cultural values tied to religious identity may influence reproductive policy preferences. Model 2, which adds racial demographics, strengthens the explanatory power of the model. Religiosity remains significant (*β* = −0.031, *p* < 0.01), and the coefficients for Black and Hispanic population percentages are positive, though marginally significant, indicating that more racially diverse states may lean slightly toward more permissive surrogacy policies.

**Table 4:** Spatial lag regression results predicting surrogacy law permissiveness across United States.

Variable	Model 1	Model 2	Model 3	Model 4
Religious	−0.024 (0.012) ∗	−0.031 (0.011) ∗∗	−0.014 (0.050)	−0.023 (0.014)
White Percent		0.020 (0.013)	0.032 (0.036)	0.031 (0.014) ∗
White ∗ Religious			−0.000 (0.001)	
Black Percent		0.023 (0.015)	0.022 (0.016)	0.029 (0.018)
Hispanic Percent		0.021 (0.012)	0.021 (0.013)	0.030 (0.016)
Political Affiliation				−0.128 (0.183)
Party Control				−0.117 (0.251)
Median Income				0.000 (0.000)
Urban Percent				−0.006 (0.007)
Unemployment Rate				−0.006 (0.122)
College Degree Percent				−0.004 (0.028)
Population				−0.000 (0.000)
Density				0.000 (0.000)
W_surrogacy	0.309 (0.446)	0.365 (0.371)	0.300 (0.220)	0.044 (0.250)
Pseudo R-squared	0.253	0.269	0.291	0.406
Spatial Pseudo R-squared	0.312	0.347	0.351	0.414
Number of Observations	50	50	50	50
Number of Variables	3	5	6	14
Degrees of Freedom	47	45	44	36
Anselin–Kelejian test	1.066 (0.301)	2.062 (0.151)	2.922 (0.087)	1.558 (0.212)

Note: Standard errors in parentheses.

∗∗∗p < 0.001, ∗∗p < 0.01, ∗p < 0.05.

Model 3 includes an interaction term between religiosity and the White population. While the interaction is not statistically significant, its inclusion helps explore whether the effect of religiosity varies by racial composition. The main effect of religiosity becomes non-significant, suggesting potential effect modification by racial context, though further research is needed due to limited statistical power. In the Full Model (Model 4), which adjusts for political affiliation, income, education, urbanization, and unemployment, the percentage of White residents remains a significant positive predictor of surrogacy permissiveness (*β* = 0.031, *p* < 0.05), while religiosity trends negatively but is no longer statistically significant. These results suggest that while religious adherence is associated with restrictive policy environments, this effect is attenuated when broader structural factors are considered. Overall, the models confirm that spatial factors, cultural attitudes, and demographic composition collectively shape state surrogacy policies. The use of spatial lag regression is crucial, as it accounts for geographic clustering, ensuring more accurate and policy-relevant estimates.

The models in Table 4 also include a spatially lagged dependent variable (**W_surrogacy**) to account for geographic diffusion effects. To address potential endogeneity between the lagged outcome and state-level predictors, we instrumented the lag term using the spatially lagged predictor (W_religious), following standard two-stage procedures in spatial econometrics. To evaluate whether additional spatial dependence remained in the model residuals, we conducted the Anselin–Kelejian test for spatial dependence. The test statistic was not significant (for example, χ^2^ = 1.066, df = 1, p = 0.302 in model 1), indicating that once the spatial lag of the dependent variable was included, there was no residual spatial autocorrelation unaccounted for in the model. This suggests that the spatial lag specification adequately captured the geographic clustering in surrogacy policy permissiveness and that the model is well-specified with respect to spatial dependence.

### 3.5 Ordered logistic regression results: associations between religiosity, race, and surrogacy policy permissiveness

To test the robustness of our findings, we estimated ordered logistic regression (ologit) models using the three-level categorical outcome of surrogacy policy permissiveness (restrictive, neutral, permissive). Table 5 reports the results of these models, which are comparable in structure to the spatial lag models presented in Table 4.

**Table 5:** Ordered logistic regression results predicting surrogacy law permissiveness across United States (N = 50).

Variable	Model 1 (Religious)	Model 2 (+Race)	Model 3 (+Interaction)	Model 4 (Full)
Religious	−0.124 (0.035) ∗∗∗	−0.167 (0.048) ∗∗∗	0.013 (0.239)	−0.123 (0.068) †
White Percent		0.116 (0.056) ∗	0.242 (0.179)	0.157 (0.068) ∗
Black Percent		0.131 (0.065) ∗	0.116 (0.067) †	0.155 (0.087) †
Hispanic Percent		0.111 (0.054) ∗	0.116 (0.054) ∗	0.158 (0.078) ∗
Religious × White %			−0.002 (0.003)	
Political Affiliation				−0.571 (0.937)
Party Control				−0.446 (1.360)
Median Income				0.00007 (0.00006)
Urban Percent				−0.036 (0.036)
Unemployment Rate				−0.088 (0.572)
College Degree %				−0.030 (0.133)
Population				−1.5e–08 (5.8e–08)
Density				0.0004 (0.0015)

Cut1	−9.41	−0.73	9.00	4.79
Cut2	−5.57	3.45	13.20	9.27
LR χ^2^	16.65 ∗∗∗	22.67 ∗∗∗	23.27 ∗∗∗	26.44 ∗∗
Pseudo R^2^	0.182	0.247	0.254	0.288

*Note:* Standard errors in parentheses. †p < 0.10, ∗p < 0.05, ∗∗p < 0.01, ∗∗∗p < 0.001.

Model 1 demonstrates that higher state religiosity is significantly associated with more restrictive surrogacy laws (β = −0.124, p < 0.001). Model 2 introduces racial composition variables, revealing that states with higher proportions of White, Black, and Hispanic residents are significantly more likely to adopt permissive surrogacy policies, while religiosity remains negatively associated with permissiveness. The ordered logistic models indicate that states with higher proportions of White residents, as well as those with larger Black and Hispanic populations, were more likely to adopt permissive surrogacy policies. This pattern suggests that permissiveness is not limited to racially homogenous states but also occurs in more racially diverse contexts, such as California and New Jersey, which combine higher diversity with supportive surrogacy laws. Model 3 adds an interaction term between religiosity and White population share, though neither the interaction nor the main effects reach statistical significance, likely due to limited statistical power. In the fully adjusted Model 4, racial composition variables (White %, Black %, and Hispanic %) remain positive and significant predictors of permissive policy adoption, whereas religiosity trends negative but falls short of conventional significance thresholds (p = 0.07).

Together, the two modeling strategies offer complementary perspectives. The spatial lag models (Table 4) highlight the role of geographic clustering and diffusion, while the ordered logistic models (Table 5) confirm that religiosity and racial composition remain consistent predictors of surrogacy policy permissiveness when explicitly modeling the outcome as ordinal. The convergence of findings across these approaches strengthens confidence in the robustness of our results.

## 4 Discussion

This study is the first to use spatial econometric techniques to examine how religiosity, racial composition, and neighboring-state effects jointly shape the patchwork of the United States surrogacy laws. Consistent with prior scholarship documenting the moral underpinnings of reproductive policy (Kimport, 2019; Andrzej Kulczycki, 2007a, 2007b), we found that higher state religiosity was strongly associated with more restrictive surrogacy statutes in bivariate and baseline spatial-lag models. However, once broader structural factors were introduced, religiosity's direct effect attenuated and lost statistical significance. This pattern suggests that religious opposition to surrogacy is partly channeled through—and conditioned by—other sociopolitical structures such as race, income, and partisan control (Alan Abramowitz & Jennifer McCoy, 2019b; Peterson & Westwood, 2020). It is important to note that the composite religiosity measure does not disaggregate denominational differences. Prior research suggests that certain traditions—particularly evangelical Protestantism and Catholicism—are more likely to oppose surrogacy and other assisted reproductive technologies, while mainline Protestant and nonreligious populations tend to be more supportive. The aggregate nature of the Pew index therefore may mask variation in how specific traditions influence policy environments. Future work could refine this measure by incorporating denominational distributions alongside overall religiosity to better capture the religious-cultural determinants of surrogacy laws.

The persistence of the White-population share as a positive predictor of legal permissiveness after full adjustment complicates the common assumption that predominantly White states reliably enact conservative reproductive regulations. Our results echo emerging evidence that racial homogeneity can, in some contexts, align with progressive reproductive policy—perhaps because White majorities in higher-income, less religious coastal states mobilize differently around family-building technologies than do similar majorities in the rural South or Midwest (Engelhardt, 2020). These results complicate the assumption that racial diversity alone predicts restrictive reproductive environments. Instead, both a higher White population share and racial diversity were associated with permissive policies, suggesting that demographic context may interact with political culture, economic resources, and advocacy networks in shaping state-level legislation.

Spatial diagnostics underscore that surrogacy policy diffuses regionally rather than emerging solely from internal state characteristics. Significant global Moran's I and the presence of Local Moran's “low-high” and “high-low” outliers point to policy learning, emulation, and boundary-spanning advocacy networks (Berry & Berry, 2018, pp. 253–297; Shipan & Volden, 2008). Colorado's high-low status, for example, likely reflects its long-standing role as a biomedical hub in a largely restrictive Mountain West. Conversely, the permissive legislation of Arizona and Alaska despite restrictive neighbors may stem from strategic court decisions and organized patient advocacy, illustrating how diffusion is neither automatic nor unidirectional.

From a public-health perspective, these findings highlight two urgent concerns. First, legal fragmentation imposes financial and psychosocial burdens on intended parents who must cross state lines, which exacerbates geographic, racial, and class disparities in access to assisted reproduction (Beilby et al., 2024; White et al., 2021). Second, because spatial clustering indicates that states seldom act in isolation, targeted advocacy in pivotal “boundary” jurisdictions may catalyze broader regional change—mirroring diffusion observed for same-sex marriage and abortion policy (Mitchell, 2018). Health agencies should therefore incorporate legal-environment indicators into service planning—recognizing surrogacy policy as a social determinant of reproductive health.

## 5 Limitations

This study has several limitations that should be acknowledged. First, the analysis is limited to a cross-sectional snapshot of surrogacy laws as of 2025. Without longitudinal data, we cannot assess the timing of adoption or capture dynamic policy shifts over time. Future studies should employ event-history or panel approaches to model temporal diffusion more directly. Specifically, incorporating temporal dynamics would allow us to test whether surrogacy policies diffuse in waves similar to other family-policy domains, such as abortion or same-sex marriage. Second, our models are constrained by the small sample size (N = 50), which limits statistical power and degrees of freedom when multiple covariates are included. This increases the risk of Type II error and likely contributes to the non-significance of some theoretically relevant predictors and interaction terms. Third, although we employed both spatial lag and ordered logistic regression models to address different aspects of the data structure, each method entails trade-offs. The spatial lag models capture geographic clustering but treat the outcome as continuous, while the ordered logistic models respect the ordinal structure of surrogacy policy but do not incorporate spatial dependence. By presenting both, we aimed to provide complementary perspectives, but neither fully integrates spatial and ordinal features simultaneously. Fourth, the composite religiosity index does not disaggregate across denominations, which may obscure differential effects of specific religious traditions on surrogacy policy attitudes. Finally, our spatial weight matrix relied on contiguity, which captures geographic proximity but not non-geographic influences such as shared political networks, litigation pathways, or digital advocacy campaigns that may also diffuse policy innovations.

## 6 Conclusion

Surrogacy policy in the United States is not merely a function of moral beliefs or partisan ideology in isolation; it is produced at the intersection of religiosity, racial context, and regional diffusion processes. Recognizing these layered determinants is essential for policymakers and public health practitioners seeking to reduce geographic inequities in access to family-building technologies. This study provides the first state-level, spatial analysis of the United States surrogacy laws, demonstrating that surrogacy policy permissiveness is not randomly distributed but clusters geographically, diffusing through regional networks of sociopolitical influence. Religiosity emerges as a key driver of restrictive policy; however, its effect is moderated by racial context and substantially attenuated once broader structural covariates are introduced. States with larger White populations and lower religiosity remain the most permissive, whereas highly religious states—particularly those in the South and Midwest—retain restrictive or ambiguous statutes. Political partisanship, while directionally consistent with theoretical expectations, becomes insignificant after accounting for demographic and socioeconomic factors, underscoring the complex interplay among culture, race, and structural conditions in reproductive policy adoption.

These findings have three public-health implications. First, legal fragmentation forces intended parents in restrictive jurisdictions to cross state lines, creating financial and psychological burdens and reinforcing geographic inequities in access to assisted reproductive technologies. Second, the demonstrated spatial spillovers suggest that policy entrepreneurs and advocates could accelerate the diffusion of permissive laws by targeting pivotal “boundary” states that neighbor both restrictive and permissive regimes. Finally, public health practitioners should integrate legal environment indicators into needs assessments for fertility and family-building services, recognizing that policy context is a social determinant of reproductive health. As surrogacy becomes an increasingly viable path to parenthood, especially for LGBTQ + families, single individuals, and those facing medical infertility, the fragmented legal landscape creates uncertainty and reinforces disparities in access. Public health policy must recognize these barriers and prioritize efforts to harmonize surrogacy regulations across states. Future research should examine how legal variation translates into real-world access, health outcomes, and family formation experiences.

This study advances the literature by combining spatial econometrics with sociopolitical theory to explain surrogacy law variation. Policymakers seeking to promote equitable access to reproductive technologies should consider not only internal state characteristics but also the powerful, regionally clustered forces of culture and demography that shape the United States surrogacy landscape.

**Limitations.** The cross-sectional design precludes causal inference and cannot capture policy changes after 2025. Although variance-inflation diagnostics mitigate multicollinearity concerns, the modest sample (N = 50) limits power to detect complex interactions. Finally, the spatial weight matrix relied on contiguity, which may not capture policy influence transmitted through digital advocacy or shared religious networks.

**Future directions.** Longitudinal approaches, such as event history or difference-in-differences designs, could assess how key court decisions or federal interventions trigger state-level policy changes over time. Mixed-methods research could deepen insight into how surrogacy policy is framed and contested by religious leaders, advocacy organizations, and fertility professionals. Cross-national spatial comparisons may also reveal whether the United States pattern mirrors those in countries with centralized health systems, such as Australia or within the European Union.

## CRediT authorship contribution statement

**Jingjing Gao:** Writing – review & editing, Writing – original draft, Visualization, Validation, Supervision, Software, Resources, Project administration, Methodology, Investigation, Funding acquisition, Formal analysis, Data curation, Conceptualization. **Muinat Abolore Idris:** Writing – review & editing. **Gabriela A. Gallegos:** Writing – review & editing. **Bryan Colby Griffin:** Data curation. **Sharon V. Munroe:** Writing – review & editing. **Jason H. Windett:** Writing – review & editing.

## Ethics statement

This study did not involve human participants, identifiable private information, or interventions. All data were obtained from publicly available secondary sources, including government databases and publicly released policy documents. As such, ethical approval and informed consent were not required. The research complies with all relevant ethical regulations for studies using non-human, de-identified, aggregated data.

## Declaration of interest

The authors declare no conflicts of interest relevant to the content of this manuscript.

## Data Availability

The data used in this study are publicly available as described in the method part.
